# Validation of the Clinical Dementia Rating scale without informant

**DOI:** 10.1002/alz70857_102368

**Published:** 2025-12-25

**Authors:** Marissa Moore, Manuel Montero‐Odasso, Osvaldo Espin‐Garcia, Frederico Pieruccini‐Faria

**Affiliations:** ^1^ University of Western Ontario, London, ON, Canada; ^2^ Gait and Brain Lab, Parkwood Institute, London, ON, Canada; ^3^ Parkwood Institute, London, ON, Canada; ^4^ Schulich School of Medicine & Dentistry, Division of Geriatric Medicine, Western University, London, ON, Canada; ^5^ Gait and Brain Laboratory, Parkwood Institute, London, ON, Canada; ^6^ Gait & Brain Lab; Lawson Research Institute; Schulich School of Medicine& Dentistry, Division of Geriatric Medicine, Western University, London, ON, Canada

## Abstract

**Background:**

There are over 55 million individuals living with dementia worldwide, reaching 10 million new cases every year according to the World Health Organization. A crucial first step is identifying individuals at risk of dementia and trying to prevent of delay the progression, since up to 40% of dementia cases are potentially preventable. Thus, tools such as the Clinical Dementia Rating scale (CDR), the gold‐standard in staging dementia, are crucial. However, the CDR requires the presence of an informant (e.g. spouse, caregiver) which is a challenge for 30% of Canadians who live alone or do not have an informant available or willing to answer questions. Hence, we have created a novel CDR version without the need of an informant, which will simplify the staging of dementia thus helping more individuals. Our study aims to validate the CDR without informant and we hypothesize a high level of agreement across the two versions.

**Method:**

The CDR without informant has been created by replacing the information obtained from the informant with goal‐oriented questions asked directly to the participant. Cognitively impaired participants (score below 26 on the Montreal Cognitive Assessment (MoCA)) (*n* = 60) already recruited from the Gait and Brain study cohort will be administered both CDR versions during their scheduled annual assessment. Intraclass correlation and interrater reliability will be calculated (correlation of at least 0.6, assuming statistical power of 0.8 and alpha of 0.05).

**Result:**

The results of this study are in progress.

**Conclusion:**

Direct assessment of an individual allows for increased opportunity to classify progression from cognitive impairment to dementia, elucidating the importance of this new tool. This novel CDR without informant can help clinicians and researchers to assess those impacted with dementia, when caregiver information is not feasible to obtain or is unreliable.

